# Systemic lupus erythematosus simultaneously presenting with visceral muscle dysmotility syndrome and mechanical intestinal obstruction clinically relieved by surgery: a case report and literature review

**DOI:** 10.1186/s12876-022-02105-3

**Published:** 2022-01-25

**Authors:** Junxian Wen, Weijie Chen, Lu Gao, Xiaoyuan Qiu, Guole Lin

**Affiliations:** 1 Department of Neurosurgery, Peking Union Medical College Hospital, Peking Union Medical College, Chinese Academy of Medical Sciences, No. 1 Shuaifuyuan, Dongcheng District, Beijing, 100730 People’s Republic of China; 2 Department of Surgery, Peking Union Medical College Hospital, Peking Union Medical College, Chinese Academy of Medical Sciences, No. 1 Shuaifuyuan, Dongcheng District, Beijing, 100730 People’s Republic of China; 3 Department of Breast Surgery, Peking Union Medical College Hospital, Peking Union Medical College, Chinese Academy of Medical Sciences, No. 1 Shuaifuyuan, Dongcheng District, Beijing, 100730 People’s Republic of China

**Keywords:** Systemic lupus erythematosus, Mechanical intestinal obstruction, Visceral muscle dysmotility, Intestinal pseudo-obstruction, Case report

## Abstract

**Background:**

Intestinal pseudo-obstruction (IPO) accompanied by hepatobiliary dilatation and ureterohydronephrosis is extremely rare in systemic lupus erythematosus (SLE). This triad is also called visceral muscle dysmotility syndrome (VMDS). Only 9 cases have been reported in the literature. Here, we report a rare case of VMDS with mechanical intestinal obstruction that was clinically relieved by surgery.

**Case presentation:**

This report refers to a 31-year-old woman with SLE and gastrointestinal symptoms presented as abdominal pain, nausea and stoppage of the passage of flatus or stool without obvious reasons. The patient suffered from severe abdominal distension because of massive flatulence. Contrast-enhanced computed tomography (CT) of the abdomen performed in our hospital showed localized stenosis of the bowel, ureterohydronephrosis, and biliary tract dilatation. Endoscopy showed a stenotic segment located in the sigmoid colon. The colon biopsy samples suggested that the stenosis was caused by inflammatory tissues. Biochemical investigations showed hypoalbuminemia, electrolyte disturbance and decreased C3. Antinuclear antibody was positive. After careful assessment, transverse colostomy was performed for this patient. Gastrointestinal symptoms were clinically relieved after the surgery.

**Conclusion:**

To the best of our knowledge, no VMDS patients have presented with mechanical ileus before. This case is the first documented occurrence of SLE with VMDS and mechanical intestinal obstruction symptoms relieved by surgery. Due to the low incidence of this condition, no standard treatment regimen has been established. However, surgical treatment offers significant benefit in specific situations.

## Background

Systemic lupus erythematosus (SLE) is a prevalent autoimmune disease which presents with various clinical features and manifestations [1]. Intestinal pseudo-obstruction (IPO) is one of the most uncommon gastrointestinal system symptoms in SLE [2]. In some rare cases, patients could present with IPO and pyeloureterectasis and biliary tract dilatation simultaneously. This rare triad is named generalized megaviscera of lupus (GML) or visceral muscle dysmotility syndrome (VMDS) [3, 4]. To date, only 9 cases have been reported in the literature [2, 3, 5, 6]. However, here, we present an even rarer case with VMDS and mechanical intestinal obstruction secondary to SLE. To our knowledge, this is the first report of an SLE patient presenting with these symptoms simultaneously.

## Case presentation

The patient was a 31-year-old woman with a 15-year history of erythema on the cheek, swelling and pain of both knees, and abdominal distension. She had a history of idiopathic thrombocytopenia verified in December 1997. The patient had been maintained on prednisone 10 mg qd. In late March 2021, the patient suddenly stopped defecating, and this was accompanied by abdominal distension, intermittent nausea and retching. An indwelling gastric tube was placed in another hospital and the patient was treated with daily enemas. Gastric tube drainage was 300–400 ml per day. In mid-April, she was prescribed methylprednisolone 500 mg × 3 d shock treatment in addition to cyclophosphamide 0.2 g iv qod. The abdominal distension was relieved, but there was still no bowel movement. Finally, the patient came to our hospital on May 6 because of paroxysmal colic in the lower abdomen.

**Fig. 1:**
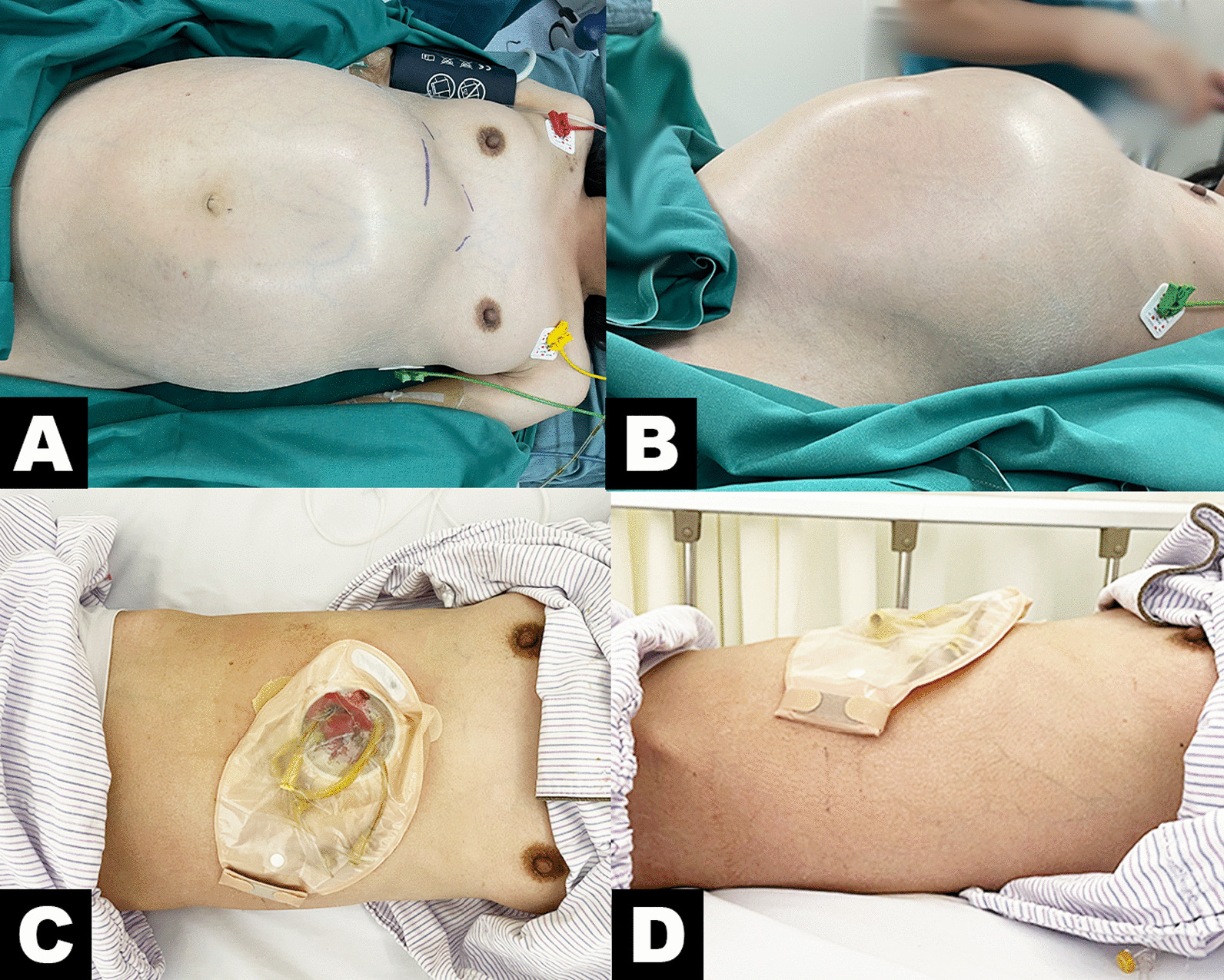
Preoperative and postoperative images of the abdomen of the patient. Top view (**A**) and lateral view (**B**) show patient’s extremely distended abdomen before surgery. The patient’s abdomen flattened after surgery as seen in top view (**C**) and lateral view (**D**)

On admission, findings from physical examination were as follows: body temperature was 36.6 °C, pulse was 84/min, respiratory rate was 18/min, and blood pressure was 127/101 mmHg. The patient entered the room in a wheelchair with a gastric tube and a right subclavian central venous catheter. The patient's abdomen was extremely distended, with tenderness in the left lower quadrant (Fig. 1) but no rebound tenderness. The bowel sounds were very weak and hardly audible. Liver and spleen were not palpable in the subcostal and subxiphoid regions.

Blood tests showed positive antinuclear antibody (1:160); anti-dsDNA antibody, (−); antiRNP, (−); anti-Sm, (−); anti-SSA, (−); anti-SSB, (−); anti-ribosomal P, (−); anti-cardiolipin antibody, (−); and lupus anticoagulant, 1.2. In immunoserological testing, CRP was 33.08 mg/l; C3, 0.538 g/l; and C4, 0.408 g/l. Biochemical testing showed serum albumin was 27 g/l; ALT 40 U/L; TBil 10.7 μmol/L; DBil 6.6umol/L; Gamma-glutamyltransferase (GGT) 433 U/L; K, 3.4 mmol/L; Na, 137 mmol/L; Ca, 2.07 mmol/L, Fe 14 μg/dL; Cr 67 μmol/L; and Urea 6.13 mmol/L. Coombs test was positive, and total urine protein was 1.64 g/24 h.

**Fig. 2:**
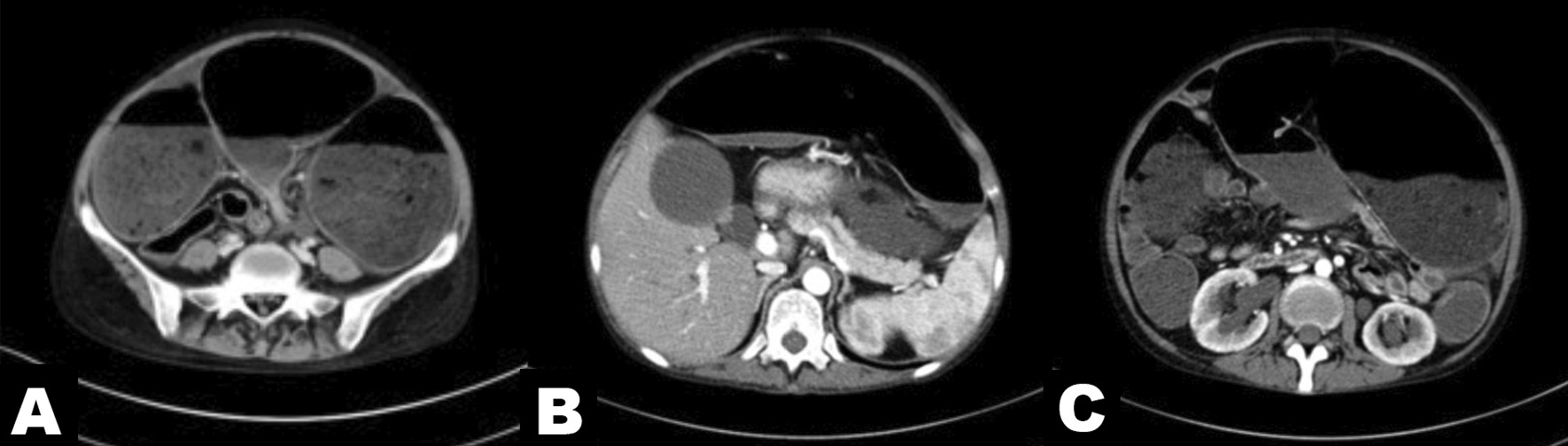
Computed tomography scans of the abdomen. Localized stenosis of the sigmoid colon and general dilation of the bowel (**A**); dilated intra- and extra-hepatic bile ducts, dilated pancreatic ducts and enlargement of the gallbladder (**B**); dilation of the renal pelvis, calyces and ureter (**C**)

**Fig. 3:**
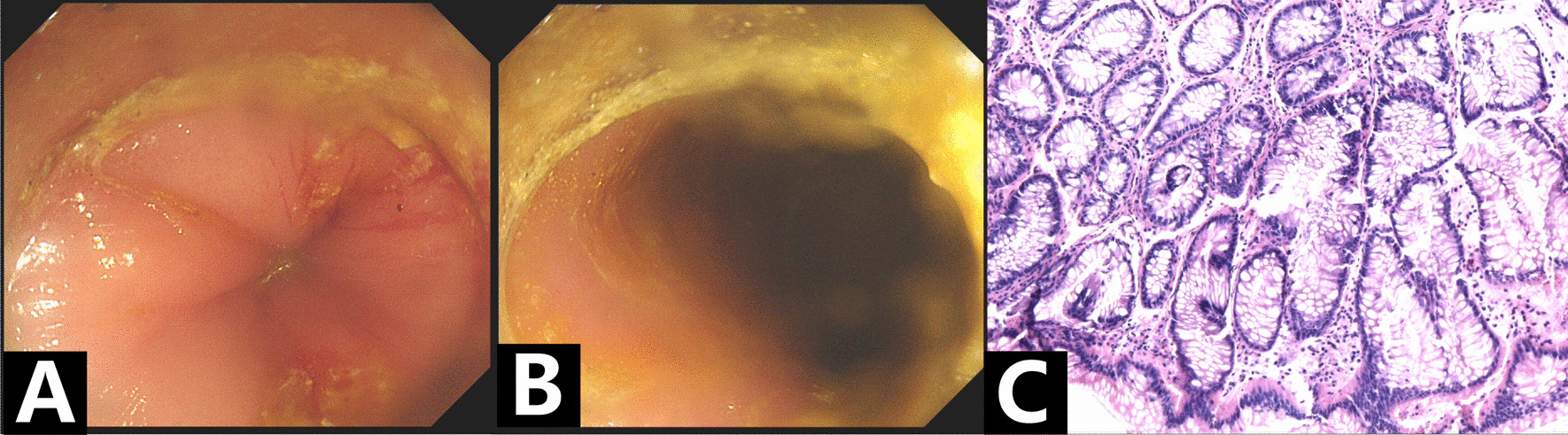
Colonoscopy and pathology pictures of sigmoid stenosis. Colonoscopy revealed a stenotic segment located 17–20 cm from the anus (**A**), with smooth local mucosa and extremely dilated anterior bowel (**B**). The pathology results of sigmoid stenosis showed chronic mucosal inflammation (**C**)

A contrast-enhanced computed tomography (CT) scan (Fig. 2) on May 28 showed general dilated small and large bowel; dilated intrahepatic and extrahepatic bile ducts and dilated pancreatic ducts; significant enlargement of the gallbladder; bilateral dilatation of the renal pelvis, calyces and ureter; and localized stenosis of the sigmoid colon with dilatation of the upper intestinal canal with fluid flattening. Colonoscopy (Fig. 3) revealed a stenotic segment located 17–20 cm from the anus, with smooth local mucosa and extremely dilated anterior bowel. The pathological results of sigmoid stenosis also showed chronic mucosal inflammation.

**Fig. 4:**
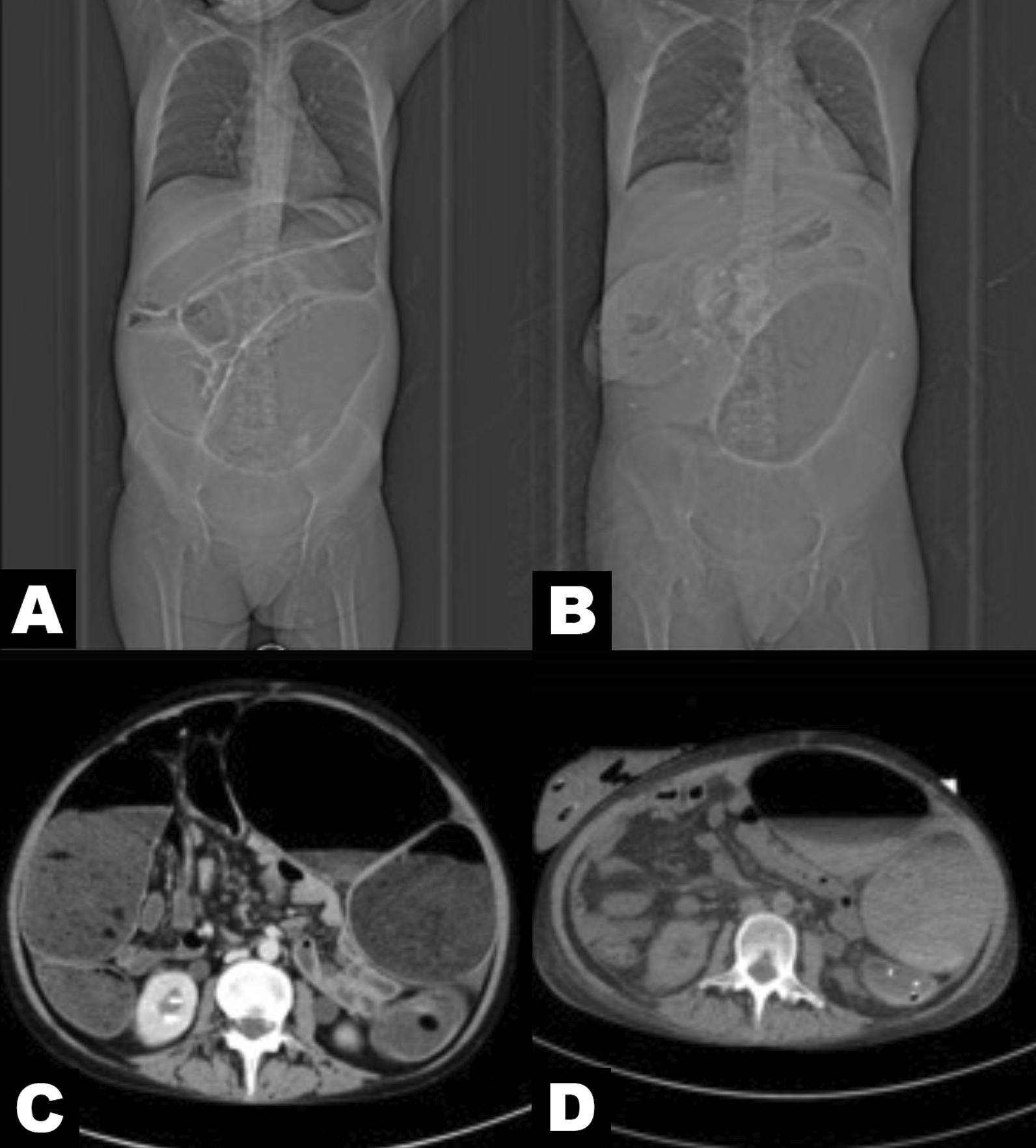
Preoperative and postoperative abdominal computed tomography scans. Compared to preoperative abdomen reconstruction (**A**) and axial reconstruction (**C**), postoperative abdomen reconstruction (**B**) and axial reconstruction (**D**) showed that the patient’s bowel dilatation had improved significantly after surgery

Four days later, a catheter was inserted through the patient's anus to relieve intestinal obstruction, but the abdominal distension did not decrease and was accompanied by abdominal pain and dyspnoea. After several multidisciplinary discussions, we performed a transverse colostomy for this patient. We aspirated approximately 3000 ml of stool contents during the operation. After the operation, the patient's abdomen flattened, and abdominal distension improved significantly (Fig. 1). CT (Fig. 4) on June 7 showed that the patient's bowel dilatation had improved significantly. Gradually, voluntary bowel movements returned and the patient started enteral nutrition. In this case, early vigorous surgical intervention reversed her deteriorating condition and thus yielded a good outcome. A half year later, we performed a follow-up examination of her symptoms of abdominal distention, and we found that her symptoms did not recur. However, this patient strongly refused any further radical surgery. The histopathology of sigmoid stenosis is still unclear.

## Discussion and conclusions

SLE is an autoimmune disease that predominantly affects women and typically has manifestations in multiple organs, including the skin, kidneys, joints, and gastrointestinal system [7]. Immune system activation in SLE is characterized by exaggerated B cell and T cell responses and loss of immune tolerance against self-antigens [8]. The prevalence of SLE in adults ranges from 30 to 150 per 100 000, and the incidence ranges from 2.2 to 23.1 per 100 000 per year [9]. Therapeutic approaches for SLE involve immunomodulation and immunosuppression, which are targeted to reduce disease activity in specific organ manifestations [1, 9]. However, despite many treatment advances, SLE continues to cause substantial morbidity and mortality.

Our case is an SLE patient who developed severe intestinal complications. IPO is an uncommon complication in SLE and is defined as ineffective intestinal propulsion with the clinical features of intestinal obstruction but without mechanical obstruction. IPO can present as an initial manifestation of SLE. The most common clinical presentation is abdominal pain, followed by nausea and/or vomiting, abdominal distension, diarrhoea and constipation. This case presented all these symptoms. Pardos-Gea et al. reported in 2005 that IPO was associated with pyeloureterectasis and biliary tract dilatation [6]. *Frederick-D *et al. and Chen et al. separately named this rare triad generalized megaviscera of lupus (GML) [5] and visceral muscle dysmotility syndrome (VMDS) [3]. According to a previous report, the incidence of VMDS in patients with SLE was less than 0.15% [2]. The underlying mechanism of VMDS might be vasculitis of the visceral smooth muscles, which leads to muscular damage and hypomotility and eventually to concurrent pyeloureterectasis or megacholedochus [10]. Many studies have shown that it can be associated with increased morbidity and mortality [11]. Early recognition of this life-threatening problem and the vigorous use of immunosuppressives can avoid unnecessary ineffective interventions and potentially fatal complications [3, 6].

In addition, this patient concurrently suffered from mechanical ileus. To our knowledge, this was never reported before. Because of the presence of sigmoid stenosis, the patient had to undergo emergency surgery. Unfortunately, although we collected tissue from the stenosis during colonoscopy and performed pathological examination, we did not find the exact cause of the stenosis. We speculate that ileus was caused by dysfunction of the visceral autonomic nervous system, which was reported in some cases [12, 13]. Because enteric neurons are essential for propulsive intestinal motility, the absence of intrinsic enteric neurons could lead to a lack of propulsive motility patterns in the distal bowel. Moreover, inhibitory enteric motor neurons mediate the relaxation of the gut wall during propulsive motility, therefore, an absence of intrinsic inhibitory neurons may also contribute to the persistent constriction of the corresponding segment [14]. We posit that dysfunction of the inhibitory neuron led to localized spastic narrowing of the patient’s sigmoid colon and resulted in dilatation of the anterior segment, as in the clinical presentation of Hirschsprung disease [15]. Unfortunately, because of the high intra-abdominal pressure and poor health of this patient, we did not remove her diseased bowel in the initial surgery, but only performed a colostomy, and our suspicions were not confirmed.

High-dose steroids and immunosuppressants are the first choice in patients with VMDS secondary to SLE. Early and vigorous immunosuppressive treatment will prevent unnecessary exploratory laparotomy and complications, while dose tapering should be slowed to avoid recurrence^3^. However, if the patient has a concurrent local intestinal obstruction, autonomic neuropathy should be considered. They should be identified early and the affected segment of the rectum and colon should be removed, or initial primary colostomy should be performed in cases of major health problems. In addition, enteric neurons could potentially be future therapeutic targets for SLE.

In conclusion, if general visceral muscle dysmotility is detected in a patient with SLE and the radiological examination suggests partial intestinal obstruction, doctors should consider the possibility that both VMDS and mechanical intestinal obstruction are present simultaneously. Although more evidence is required to determine the optimal treatment choice, resection of the obstructed bowel or colostomy as an initial treatment is recommended.

## Data Availability

All information about the patient came from the Department of Rheumatology and Department of Surgery, Peking Union Medical College Hospital.

## Abbreviations

IPO: Intestinal pseudo-obstruction
SLE: Systemic lupus erythematosus
VMDS: Visceral muscle dysmotility syndrome
CT: Computed tomography
